# Comparison of Clinical Characteristics Among COVID-19 and Non-COVID-19 Pediatric Pneumonias: A Multicenter Cross-Sectional Study

**DOI:** 10.3389/fcimb.2021.663884

**Published:** 2021-07-01

**Authors:** Zhongwei Jia, Xiangyu Yan, Liwei Gao, Shenggang Ding, Yan Bai, Yuejie Zheng, Yuxia Cui, Xianfeng Wang, Jingfeng Li, Gen Lu, Yi Xu, Xiangyu Zhang, Junhua Li, Ning Chen, Yunxiao Shang, Mingfeng Han, Jun Liu, Hourong Zhou, Cen Li, Wanqiu Lu, Jun Liu, Lina Wang, Qihong Fan, Jiang Wu, Hanling Shen, Rong Jiao, Chunxi Chen, Xiaoling Gao, Maoqiang Tian, Wei Lu, Yonghong Yang, Gary Wing-Kin Wong, Tianyou Wang, Runming Jin, Adong Shen, Baoping Xu, Kunling Shen

**Affiliations:** ^1^ School of Public Health, Peking University, Beijing, China; ^2^ Center for Intelligent Public Health, Institute for Artificial Intelligence, Peking University, Beijing, China; ^3^ Center for Drug Abuse Control and Prevention, National Institute of Health Data Science, Peking University, Beijing, China; ^4^ China National Clinical Research Center for Respiratory Diseases, Department of Respiratory Medicine, Beijing Children’s Hospital, National Center for Children’s Health, Capital Medical University, Beijing, China; ^5^ Research Unit of Critical Infection in Children, Chinese Academy of Medical Sciences, Beijing, China; ^6^ Department of Pediatrics, The First Affiliated Hospital of Anhui Medical University, Hefei, China; ^7^ Pediatric Department, Union Hospital, Tongji Medical College, Huazhong University of Science And Technology, Hubei, China; ^8^ Department of Respiratory Medicine, Shenzhen Children’s Hospital, Shenzhen, China; ^9^ Department of Pediatrics, Guizhou Provincial People’s Hospital, Guizhou, China; ^10^ Department of Pediatrics, Shenzhen Third People’s Hospital, Second Affiliated Hospital of Southern University of Science and Technology, Shenzhen, China; ^11^ Department of Pediatrics, Taihe Hospital, Shiyan, China; ^12^ Department of Pediatric, Guangzhou Women and Children’s Medical Center, Guangzhou Medical University, Guangzhou, China; ^13^ Department of Pediatrics, Xiangyang Central Hospital, Xiangyang, China; ^14^ Department of Pediatric, Shengjing Hospital of China Medical University, Liaoning, China; ^15^ Department of Respiratory Medicine, The Second People’s Hospital of Fuyang, Anhui, China; ^16^ Department of Pediatrics, The People Hospital of Bozhou, Anhui, China; ^17^ Department of General Practice, Guizhou Provincial People’s Hospital, Guiyang, Guizhou, China; ^18^ Office of Academic Research, Jiangjunshan Hospital, Guizhou, China; ^19^ Department of Pediatrics, The Affiliated Hospital of Guizhou University, Guizhou, China; ^20^ Department of Pediatrics, Jingzhou First People’s Hospital, Jingzhou, China; ^21^ Department of Pediatrics, Huangshi Maternity and Child Health Care Hospital, Huangshi, China; ^22^ Department of Pediatrics, Suizhou Maternity and Child Health Care Hospital, Suizhou, China; ^23^ Department of Pediatrics, Xiangyang First People’s Hospital, Xiangyang, China; ^24^ Department of Pediatrics, Xishui People’s Hospital, Huanggang, China; ^25^ Department of Pediatrics, People’s Hospital of Tuanfeng County, Huanggang, China; ^26^ Department of Pediatrics, Tongren People’s Hospital of Guizhou Province, Guizhou, China; ^27^ Department of Pediatrics, Yichang Central People’s Hospital, Yichang, China; ^28^ Beijing Pediatric Research Institute, Beijing, China; ^29^ Beijing Children's Hospital, National Center for Children’s Health, Capital Medical University, Beijing, China; ^30^ Department of Pediatrics, Prince of Wales Hospital, Chinese University of Hong Kong, Hong Kong Special Administrative Region, China; ^31^ Center of Hematologic Oncology, Beijing Children’s Hospital, National Center for Children’s Health, Capital Medical University, Beijing, China; ^32^ Beijing Key Laboratory of Pediatric Respiratory Infection Diseases, Beijing Pediatric Research Institute, Beijing Children’s Hospital, National Center for Children’s Health, Capital Medical University, Beijing, China

**Keywords:** COVID-19 pneumonia, viral pneumonia, non-viral pneumonia, pediatric patients, clinical characteristics

## Abstract

**Background:**

The pandemic of Coronavirus Disease 2019 (COVID-19) brings new challenges for pediatricians, especially in the differentiation with non-COVID-19 pneumonia in the peak season of pneumonia. We aimed to compare the clinical characteristics of pediatric patients with COVID-19 and other respiratory pathogens infected pneumonias.

**Methods:**

We conducted a multi-center, cross-sectional study of pediatric inpatients in China. Based on pathogenic test results, pediatric patients were divided into three groups, including COVID-19 pneumonia group, Non-COVID-19 viral (NCV) pneumonia group and Non-viral (NV) pneumonia group. Their clinical characteristics were compared by Kruskal-Wallis H test or chi-square test.

**Results:**

A total of 636 pediatric pneumonia inpatients, among which 87 in COVID-19 group, 194 in NCV group, and 355 in NV group, were included in analysis. Compared with NCV and NV patients, COVID-19 patients were older (median age 6.33, IQR 2.00-12.00 years), and relatively fewer COVID-19 patients presented fever (63.2%), cough (60.9%), shortness of breath (1.1%), and abnormal pulmonary auscultation (18.4%). The results were verified by the comparison of COVID-19, respiratory syncytial virus (RSV) and influenza A (IFA) pneumonia patients. Approximately 42.5%, 44.8%, and 12.6% of the COVID-19 patients presented simply ground-glass opacity (GGO), simply consolidation, and the both changes on computed tomography (CT) scans, respectively; the proportions were similar as those in NCV and NV group (p>0.05). Only 47.1% of COVID-19 patients had both lungs pneumonia, which was significantly lower than that proportion of nearly 80% in the other two groups. COVID-19 patients presented lower proportions of increased white blood cell count (16.5%) and abnormal procalcitonin (PCT) (10.7%), and a higher proportion of decreased lymphocyte count (44.0%) compared with the other two groups.

**Conclusion:**

Majority clinical characteristics of pediatric COVID-19 pneumonia patients were milder than non-COVID-19 patients. However, lymphocytopenia remained a prominent feature of COVID-19 pediatric pneumonia.

## Introduction

Since December 2019, cases of pneumonia with no clear etiology were reported in Wuhan, Hubei, China, which was known as the Coronavirus Disease 2019 (COVID-19) and the virus was named as severe acute respiratory syndrome coronavirus 2 (SARS-CoV-2) by the World Health Organization (WHO). It subsequently has spread rapidly to the whole world (Huang et al., 2020; Lu et al., 2020; Zhu et al., 2020 ). People of all ages are susceptible to COVID-19. Children are paid more attention as a vulnerable group. Fortunately, compared with the adult patients, the number of pediatric patients were lower and mostly with milder symptoms and better prognosis (CDC COVID-19 Response Team, 2020; National Health Commission of the People’s Republic of China, 2020). Although most pediatric patients with COVID-19 had a good prognosis, severe or critical cases or even death cases were reported (Lu et al., 2020). Additionally, with the pandemic of COVID-19 in the world, more and more pediatric cases were reported and it brings a new challenge for pediatricians.

Pneumonias are the major cause of death among infectious diseases in children less than five years old in the world, killing approximately 800,000 children a year (Liu et al., 2016). In clinical, pediatricians usually diagnosed pneumonia according to clinical manifestations and (or) chest imaging exam. Yet, fever, cough with or without sputum, wheeze are common symptoms in most cases with pneumonia even if they had different pathogens. Previous study of COVID-19 pneumonia also reported that the common symptoms were fever, cough and fatigue (Lu et al., 2020). Different from adults, 45.5% pediatric patients were afebrile, and mostly with low to moderate fever (Huang et al., 2020). The differentiation of pediatric patients with COVID-19 pneumonia and non-COVID-19 pneumonia (especially non-COVID-19 viral pneumonia) is difficult. Especially in the peak season of pneumonia during the epidemic period of COVID-19, it is a very urgent need to identify and protect children earlier. Therefore, in this study, we aim to compare the different clinical aspects of COVID-19 pneumonia and non-COVID-19 pneumonia.

## Methods

### Study Design and Participants

We conducted a multi-center, cross-sectional study of pediatric pneumonia inpatients based on retrospective collection of clinical medical records from 30 hospitals in four provinces of China, including Anhui, Guangdong, Guizhou, and Hubei (non-Wuhan cities), between January 16, 2020, and February 25, 2020 (**Supplementary Figure 1**). The time period was chosen because after that, the pediatric COVID-19 patients were rare in the four provinces. All pediatric inpatients with pneumonia of these hospitals were included in this study if patients met the following four criteria (Hu and Jiang, 2002; Shen et al., 2020): (1) ≤ 18 years old; (2) with at least one of the respiratory symptoms, such as fever, cough, sputum production, shortness of breath, and so on; (3) with wet crackles in pulmonary auscultation and/or imaging alterations in chest computed tomography (CT) scans, including obvious consolidation and/or ground-glass opacity (GGO) in the lung; (4) had pathogenic detection results. CT scan is not the routine examination for patients with pneumonia, but in this early phase of pandemic the participant centers chose to perform chest CT for every suspected COVID-19 patients in order to prevent missed diagnosis. At that emergency period, suspected COVID-19 patients were kept isolation in hospitals, and report of the confirmed PCR test needed several days after the sampling, therefore chest CT was recommended for early clinical diagnosis (Shen et al., 2020). The necessity for chest CT test was explained to children’s parents or other guardians, and informed consent was obtained from them. In order to compare the COVID-19 patients’ imaging alterations with non-COVID-19 pneumonia patients, the CT scans were also collected from non-COVID-19 pneumonia patients’ clinical medical records.

Pathogen detections were conducted for all of the pediatric inpatients, including: (1) real-time reverse-transcriptase-polymerase-chain-reaction (RT-PCR) assay of nasal and pharyngeal swab specimens for SARS-CoV-2, which was available to all of the hospitals included in this study from the beginning of the study period; (2) direct immunofluorescence assay of the respiratory virus antigen of sputum, including respiratory syncytial virus (RSV), human parainfluenza viruses (HPIVs), human metapneumovirus (HMPV), coronavirus (CoV), and human rhinovirus (HRV), adenovirus (ADV), influenza A and B (IFA,IFB), and human Bocavirus (HBoV). (3) serologic test for detecting *Mycoplasma pneumoniae* (*M. pneumonia*) antibody by passive agglutination (PA) method (Serodia-Myco II, Fujirebio, Japan). (4) bacterial cultures were prepared from secretions of lower respiratory tracts. Based on their pathogenic test results, the patients were divided into three groups, including: (1) COVID-19 pneumonia group, which was defined as patients who had positive results of SARS-CoV-2 test, and without other pathogens infection; (2) Non-COVID-19 viral (NCV) pneumonia group, which was defined as patients who had negative results of SARS-CoV-2 test, and showed other respiratory virus infection; (3) Non-viral (NV) pneumonia group, which was defined as patients who were negative in both SARS-CoV-2 and other virus, and/or detected with *M. pneumonia* and/or bacteria.

The study was approved by Beijing Children’s Hospital Ethics Committee (2020-K-20). Written informed consent was waived by the Ethics Commission for emerging infectious diseases.

### Data Collection

We obtained patients’ demographic, clinical, radiographic and laboratory test data from patients’ electronic medical records when they were on admission. A standardized data collection form was designed by the research team, and two trained physicians used the form to collect data from the 30 hospitals of the four provinces. In addition, typical CT scans of the three kinds of pneumonia were also collected. All the data collected were double-checked by two researchers.

### Measures

#### Demographic Characteristics

Demographic characteristics of the patients included their age (grouped as <1, ≥1 to <3, ≥3 to <6, ≥6 to <12, ≥12 years), and sex (male, female).

#### Clinical Symptoms and Signs

Whether patients had fever (>37.3°C) (Hu and Jiang, 2002), the duration of fever, cough, sputum production, shortness of breath, muscle ache, and gastrointestinal symptoms were recorded. According to their results of pulmonary auscultation, five categories were divided: normal, wet crackles in the lung, wheeze, rough sound, and mixing two or more abnormalities.

#### Radiographic Characteristics

The location of the pneumonia was categorized as unilateral left lung, unilateral right lung, or both lungs. Image changes of CT scans were divided into three categories, including GGO, consolidation, and both of the two changes. Pleural effusion (yes or no) was also recorded.

#### Laboratory Test Characteristics

Seven laboratory tests of blood were included in this study, and they were categorized based on the normal ranges, respectively: (1) White blood cell count (Categories: Normal, Decreased, Increased; Normal range: (4–10) ×10^9^/L)). (2) Lymphocyte count (Categories: Normal, Decreased, Increased; Normal range: (2.9–8.8)×10^9^/L (<3 months), (3.6-8.8)×10^9^/L (4 months to 8 months), (2.1–8.2)×10^9^/L (9 months to 2 years), (2.0-2.7)×10^9^/L (3–18years)) (Hu and Jiang, 2002). (3) C-reactive protein (CRP) (Categories: Normal, Abnormal; Normal range: <8 mg/L). (4) Procalcitonin (PCT) (Categories: Normal, Abnormal; Normal range: <0.25ng/mL) (5) Creatine kinase MB (CKMB) (Categories: Normal, Abnormal; Normal range: < 25 U/L). (6) Alanine aminotransferase (ALT) (Categories: Normal, Abnormal; Normal range: ≤ 40 U/L). (7) Aspartate transaminase (AST) (Categories: Normal, Abnormal; Normal range: ≤ 40 U/L).

### Statistical Analysis

Continuous variables were expressed as median (IQR) because they were not normally distributed and compared with the Kruskal-Wallis H test. Categorical variables were expressed as count (%) and compared by chi-square test or Fisher’s exact test when the data were limited. In addition to compare different clinical characteristics among the COVID-19, NCV, and NV groups, we also compare the characteristics among the COVID-19, single infection with RSV, and single infection with IFA patients for sensitivity analysis to verify the findings between COVID-19 and NCV patients. Another sensitivity analysis was conducted to adjust patients’ demographic characteristics using multivariable logistic regressions and to further verify the comparison of patients’ characteristics among COVID-19, NCV, and NV group.

A two-sided α of less than 0.05 was considered statistically significant. Considering the multiple hypothesis testing, Bonferroni correction was used when comparing characteristics between every two groups. Statistical analyses were done with SPSS, version 21.0 (IBM Corp).

## Results

### Demographic Characteristics

**Figure 1:**
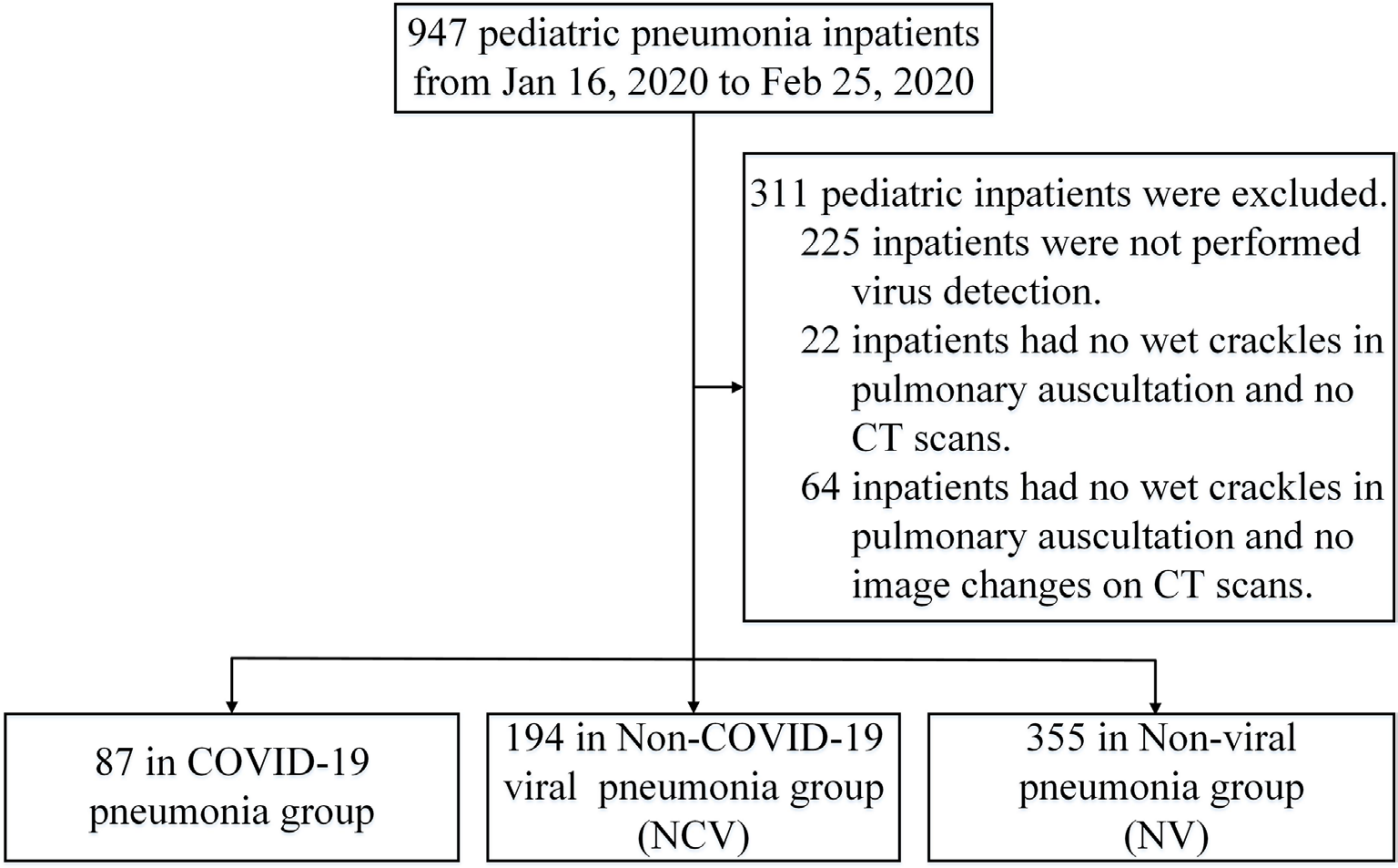
Study profile.

From January 16, 2020, to February 25, 2020, 947 pediatric pneumonia inpatients were found in the 30 hospital of the four provinces. After excluding 311 who did not meet the inclusion criteria, a total of 636 pediatric pneumonia inpatients were included in this study, including 87 in the COVID-19 group, 194 in NCV group, and 355 in NV group, respectively (**Figure 1**).

In NCV group, pediatric patients with single virus infection were accounting for 84.5% (164/194), and RSV (30.4%, 59/194) and IFA (16.0%, 31/194) were the most two common causes. The others were with mixed virus infection, approximately 15.5% (30/194), and co-infection of RSV and HBoV was predominating (7.2%, 14/194) (**Supplementary Table 1**). In NV group, 99 (27.9%) pediatric patients were infected by *M. pneumonia*, 71 (20.0%) pediatric patients were detected by single bacteria, and 30 (8.5%) pediatric patients were with mixed pathogens (19 with *M. pneumonia* and bacteria, 6 with bacteria and virus, 4 with *M. pneumonia* and virus, 1 with two type of bacteria).

The median age of all these patients were 1.83 (IQR 0.67-4.00) years. Patients in COVID-19 group (median 6.33 years, IQR 2.00-12.00 years) were significantly older than the other two groups (NCV: median 1.17 years, IQR 0.54-2.84 years, NV: median 1.75 years, IQR 0.67-3.67 years, p<0.001), and 23(26.4%) of the patients in COVID-19 groups were older than 12 years old (**Table 1**). Similarly, the age of COVID-19 patients (median 6.33 years, IQR 0.25-1.50 years) was significantly older than patients infected by RSV and IFA (RSV: median 0.75 year, IQR 0.25-1.50 years, IFA: median 1.58 years, IQR 1.00-3.00 years, p<0.001) which were the two main types of non-COVID-19 viruses (**Supplementary Table 2**). 49 (56.3%) patients in COVID-19 group were female, the proportion was higher than that in NV (37.7%, 134/355) group (**Table 1**).

**Table 1:** Demographic and clinical characteristics of patients among the three pneumonia groups.

Characteristics		Total (N = 636)	COVID-19 (N = 87)	NCV^a^ (N = 194)	NV^b^(N = 355)	P value
**Demographics**						
Age (years)						
median(IQR) ^c,d^		1.83 (0.67-4.00)	6.33 (2.00-12.00)	1.17 (0.54-2.84)	1.75(0.67-3.67)	<0.001
	<1^c,d^	197/635 (31.0)	12 (13.8)	67/193 (34.7)	118(33.2)	0.001
	≥1 to <3^c,d^	216/635 (34.0)	16 (18.4)	80/193 (41.5)	120(33.8)	0.001
	≥3 to <6	121/635 (19.1)	13 (14.9)	39/193 (20.2)	69(19.4)	0.562
	≥6 to <12^c,d,e^	75/635 (11.8)	23 (26.4)	7/193 (3.6)	45(12.7)	<0.001
	≥12 ^c,d^	26/635 (4.1)	23 (26.4)	0/193 (0.0)	3(0.8)	<0.001
Female^d^		263 (41.4)	49 56.3)	80 (41.2)	134(37.7)	0.007
**Clinical symptoms and signs**						
Fever^c,e^		443/635 (69.8)	55 (63.2)	156/193 (80.8)	232(65.4)	<0.001
Duration of Fever (days)^d^ [median(IQR)]		5.00 (2.00-7.00)	3.00 (1.00-6.00)	4.00 (2.00-7.00)	5.00(3.00-8.00)	0.007
Cough^c,d^		587 (92.3)	53 (60.9)	192 (99.0)	342(96.3)	<0.001
Sputum production^c,d,e^		367 (57.7)	30 (34.5)	138 (71.1)	199(56.1)	<0.001
Shortness of breath^c,d^		114 (17.9)	1 (1.1)	47 (24.2)	66(18.6)	<0.001
Muscle ache		2/633 (0.3)	0/85 (0.0)	0 (0.0)	2/354(0.6)	0.657
Gastrointestinal symptoms		84/634 (13.2)	11/85 (12.9)	22 (11.3)	51(14.4)	0.604
Pulmonary auscultation						
	Normal^c,d^	172 (27.0)	71 (81.6)	45 (23.2)	56(15.8)	<0.001
	Wet crackles^c,d^	225 (35.4)	8 (9.2)	66 (34.0)	151(42.5)	<0.001
	Wheeze	29 (4.6)	1 (1.1)	11 (5.7)	17(4.8)	0.232
	Rough sound with sputum	67 (10.5)	6 (6.9)	18 (9.3)	43(12.1)	0.289
	Mixing abnormalities^c,d^	143 (22.5)	1 (1.1)	54 (27.8)	88(24.8)	<0.001

a NCV, Non-COVID-19 viral pneumonia.

b NV, Non-viral pneumonia.

c Significant difference was shown between COVID-19 group and NCV group.

d Significant difference was shown between COVID-19 group and NV group.

e Significant difference was shown between NCV group and NV group.

### Clinical Symptoms and Signs

On admission, cough (92.3%, 587/636), fever (69.8%, 443/635), and sputum production (57.7%, 367/636) were three most common symptoms of pediatric pneumonia inpatients. Patients in NCV group (80.8%, 156/193) had the highest proportion of fever occurrence compared with COVID-19 (63.2%, 55/87) and NV (65.4%, 232/355) group (p<0.001) (**Table 1**). The median duration of patients’ fever in COVID-19 group was 3.00 days (IQR 1.00-6.00 days), which was significantly shorter than that in NV group (median 5.00 days, IQR 3.00-8.00 days), and had no significant difference compared with NCV group (median 4.00 days, IQR 2.00-7.00 days) (**Table 1**). Compared with NCV and NV group, COVID-19 patients were less likely to have cough (60.9%, 53/87), sputum production (34.5%, 30/87), and shortness of breath (1.1%, 1/87) symptoms (p<0.001) (**Table 1**). In COVID-19 patients, no patients had muscle ache and eleven (12.9%) patients had gastrointestinal symptoms, which had no significant differences compared with the other two groups (p>0.05) (**Table 1**). In the results of pulmonary auscultation, the proportion of normal results among COVID-19 patients (81.6%, 71/87) was 3.5 times higher than the other two groups (p<0.001); and the simply wet crackles were more likely to be found in NV group (42.5%, 151/355) compared with COVID-19 patients (9.2%, 8/87) (**Table 1**). In addition, none of the 636 patients died in hospital, all of them had been successful treated and discharged. The above results were verified after adjusting age and sex of these patients (**Supplementary Table 4**). Similar findings were also shown in the comparison of COVID-19 with RSV and IFA patients, the proportions of patients who presented with the cough, sputum production and shortness of breath in COVID-19 group were lower than that in the RSV and IFA patients (p<0.05), while the proportion of fever in COVID-19 group was only significantly lower than IFA patients (96.8%, 30/31, p<0.05), and the durations of fever among the three groups were similar (p>0.05) (**Supplementary Table 2**).

### Radiographic Characteristics

Eighteen (20.7%) of the COVID-19 patients had unilateral left lung pneumonia, 28 (32.2%) of them had unilateral right lung, and 41 (47.1%) of them had both lungs pneumonia. The proportion of both lungs pneumonia in COVID-19 patients was more than 1.5 times lower than that in NCV (80.7%, 138/171) and NV group (74.2%, 210/283, p<0.001) (**Table 2**). After adjusting age and sex, consistent results showed patients in NCV group and NV group were more likely to present both lungs pneumonia compared with COVID-19 patients (Adjusted OR(AOR)_NCV vs COVID-19_: 3.23, 95%CI: 1.76-5.91, p<0.001; AOR_NV vs COVID-19_: 2.57, 95%CI: 1.50-4.38, p=0.001) (**Supplementary Table 4**). Relatively older children were less likely to have both lungs’ pneumonia (AOR_Total_: 0.92, 95%CI: 0.85-0.99, p<0.05), and a higher proportion of patients with abnormal pulmonary auscultation had both lungs’ pneumonia compared with those who did not present abnormal pulmonary auscultation, especially for patients with wet crackles and mixing abnormalities (wet crackles: AOR_Total_ 2.56, 95%CI 1.34-4.87, p<0.01; AOR_NV_ 3.72, 95%CI 1.35-10.20, p<0.05. mixing abnormalities: AOR_Total_ 3.07, 95%CI 1.24-7.60, p<0.05; AOR_NCV_ 3.63, 95%CI 1.03-12.75, p<0.05; AOR_NV_ 3.88, 95%CI 1.11-13.53, p<0.05) (**Supplementary Table 5**). And the COVID-19 patients were also less likely to be both lung involved compared the RSV (93.8%, 45/48) and IFA (69.6%, 16/23) patients (p<0.001) (**Supplementary Table 3**).

**Table 2:** Radiographic characteristics and laboratory findings of patients among the three pneumonia groups.

Characteristics		Total (N = 636)	COVID-19 (N = 87)	NCV^a^ (N = 194)	NV^b^ (N = \355)	P value
**Radiographic characteristics**						
Location						
	Unilateral left lung^c,d^	61/541 (11.3)	18 (20.7)	13/171 (7.6)	30/283 (10.6)	0.006
	Unilateral right lung^c,d^	91/541 (16.8)	28 (32.2)	20/171 (11.7)	43/283 (15.2)	<0.001
	Both lungs^c,d^	389/541 (71.9)	41 (47.1)	138/171 (80.7)	210/283(74.2)	<0.001
Image changes CT scans						
	GGO^e^	157/351 (44.7)	37 (42.5)	72/128 (56.3)	48/136 (35.3)	0.003
	Consolidation	159/351 (45.3)	39 (44.8)	49/128 (38.3)	71/136 (52.2)	0.075
	Both changes	35/351 (10.0)	11 (12.6)	7/128 (5.5)	17/136 (12.5)	0.103
Pleural effusion		19/586 (3.2)	0/85 (0.0)	8/189 (4.2)	11/312 (3.5)	0.152
**Laboratory test**						
White blood cell count						
	median(IQR)[×10^9^/L]^c,d^	8.90 (6.41-12.12)	6.27 (4.75-8.35)	9.29 (6.98-12.29)	9.34 (6.79-12.51)	<0.001
	Decreased^c,d^	24/633 (3.8)	8/85 (9.4)	5 (2.6)	11/354 (3.1)	0.023
	Increased^c,d,e^	293/633 (46.3)	14/85 (16.5)	84 (43.2)	195/354 (55.1)	<0.001
Lymphocyte count						
	median(IQR)[×10^9^/L]^c,d^	3.56 (2.23-5.48)	2.48 (1.74-3.96)	3.65 (2.46-5.78)	3.78 (2.36-5.61)	<0.001
	Decreased^c,d^	132/562 (23.5)	37/84 (44.0)	36/181 (19.9)	59/297 (19.9)	<0.001
	Increased	113/562 (20.1)	21/84 (25.0)	38/181 (21.0)	54/297 (18.2)	0.363
CRP						
median(IQR)[ mg/L]		5.00 (1.09-15.90)	3.37 (0.81-11.48)	4.35 (1.00-15.23)	5.00 (1.12-17.20)	0.061
Abnormal		222/589 (37.7)	20/72 (27.8)	69/182 (37.9)	133/335 (39.7)	0.166
PCT						
median(IQR)[ ng/mL]^c,d^		0.16 (0.10-0.42)	0.05 (0.02-0.10)	0.19 (0.10-0.56)	0.20 (0.12-0.48)	<0.001
Abnormal^c,d^		130/339 (38.3)	6/56 (10.7)	61/134 (45.5)	63/149 (42.3)	<0.001
CKMB						
median(IQR)[ U/L]^d,e^		21.00 (3.60-32.00)	13.00 (6.38-21.25)	11.50 (1.38-25.25)	25.00 (17.00-37.00)	<0.001
Abnormal^d,e^		230/579 (39.7)	12/70 (17.1)	53/186 (28.5)	165/323 (51.1)	<0.001
ALT						
median(IQR)[ U/L]^d^		18.00 (13.00-27.00)	14.00 (11.00-25.00)	18.00 (13.00-24.00)	18.00 (13.00-28.00)	0.019
Abnormal		50/590 (8.5)	7/73(9.6)	15/187 (8.0)	28/330 (8.5)	0.920
AST						
median(IQR)[ U/L]^c,d^		35.00 (28.00-43.00)	31.20 (21.00-40.28)	36.00 (30.00-46.00)	35.00 (29.00-43.00)	0.005
Abnormal		191/590 (32.4)	18/74 (24.3)	68/188 (36.2)	105/328 (32.0)	0.178

a NCV, Non-COVID-19 viral pneumonia.

b NV, Non-viral pneumonia.

c Significant difference was shown between COVID-19 group and NCV group.

d Significant difference was shown between COVID-19 group and NV group.

e Significant difference was shown between NCV group and NV group.

**Figure 2:**
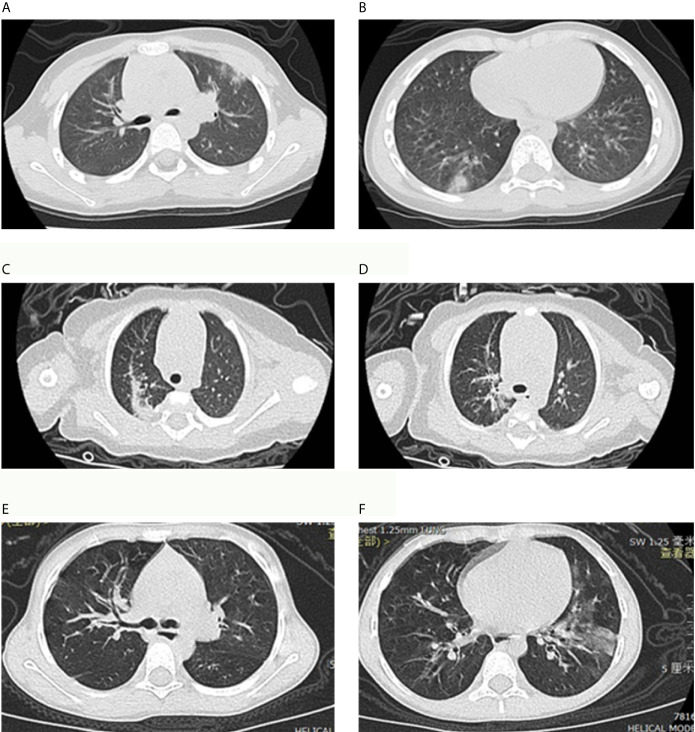
Chest computed tomography (CT) scans for COVID-19 pneumonia patient and Non-COVID-19 viral pneumonia patients. **(A, B)** showed ground-glass opacities were located in the right lower lobe of the lung and upper lobe of the left lung and located under the pleura in a patient with COVID-19 pneumonia. **(C, D)** showed multiple patchy shadows and consolidation in two lungs in a patient with respiratory syncytial virus (RSV) pneumonia. **(E, F)** showed thickened tracheobronchial wall in right lung and ground-glass opacities were located in the left lower lobe of the lung in a patient with influenza A (IFA) pneumonia.

All of the COVID-19 patients had image changes on CT scans, simply GGO were found in 37(42.5%) of them, simply consolidation were found in 39 (44.8%) of them, and eleven (12.6%) of them had the both changes (**Table 2** and **Figure 2**). The proportions of different image changes in COVID-19 patients were not significantly different with that in NCV and NV patients, which was verified by multivariable logistic regressions (**Table 2** and **Supplementary Table 4**). And similar results were also shown in the comparison of COVID-19 patients with RSV and IFA patients (**Supplementary Table 3**). Another finding was that patients with shortness of breath were more likely to present GGO on CT scans (OR_Total_: 2.36, 95%CI: 1.24-4.88, p<0.01), and among COVID-19 patients, older children were more likely to present GGO (OR_COVID-19_: 1.10, 95%CI: 1.01-1.20, p<0.05) (**Supplementary Table 6**).

Pleural effusion was a less common sign of these pneumonia patients. None of the COVID-19 patients was diagnosed with pleural effusion, and in which the three group had no significant differences (p>0.05) (**Table 2**).

### Laboratory Test Characteristics

On admission, the white blood cell count of COVID-19 patients (median 6.27 ×10^9^/L, IQR 4.75-8.35 ×10^9^/L) was almost 1.5 times lower than that in NCV (median 9.29 ×10^9^/L, IQR 6.98-12.29 ×10^9^/L) and NV patients (median 9.34 ×10^9^/L, IQR 6.79-12.51 ×10^9^/L, p<0.001). Eight (9.4%) of the COVID-19 patients had decreased white blood cell count, the proportion was over three times greater than that in NCV (2.6%, 5/194) and NV (3.1%, 11/354) group, while the proportion of increased white blood cell in COVID-19 patients (16.5%, 14/85) was almost three times lower than that in other two groups (NCV: 43.2%, 84/194, NV: 55.1%, 195/354, p<0.001) (**Table 2**). Lymphocyte count of COVID-19 patients (median 2.48 ×10^9^/L, IQR 1.74-3.96 ×10^9^/L) was also almost 1.5 times lower than that in NCV (median 3.65×10^9^/L, IQR 2.46-5.78 ×10^9^/L) and NV patients (median 3.78 ×10^9^/L, IQR 2.36-5.61 ×10^9^/L, p<0.001). In COVID-19 group, higher proportion of patients (44.0%, 37/84) occurred to be lymphocyte count decreased compared with the other two groups (p<0.001) (**Table 2**). And only ten (11.8%) of COVID-19 patients presented a lymphocyte count less than 1.5×10^9^/L. Consistently, the proportions of patients presented decreased lymphocyte count in NCV and NV group were less than COVID-19 patients (AOR_NCV vs COVID-19_: 0.50, 95%CI: 0.27-0.91, p=0.023; AOR_NV vs COVID-19_: 0.45, 95%CI: 0.26-0.78, p=0.004) (**Supplementary Table 4**). The comparison of COVID-19, RSV, and IFA patients showed that the differences among them in white blood cell count and lymphocyte count were similar to the results of comparison between COVID-19 and NCV patients (**Supplementary Table 3**).

There were no significant differences in CRP results among the COVID-19, NCV and NV group (P>0.05). While, PCT of COVID-19 patients (median 0.05 ng/mL, IQR 0.02-0.10 ng/mL) was almost four times lower than the other two groups (NCV: median 0.19 ng/mL, IQR 0.10-0.56 ng/mL, NV: median 0.20 ng/mL, IQR 0.12-0.48 ng/mL, p<0.001). And the proportion of patients whose PCT tests were abnormal in COVID-19 group (10.7%, 6/56) was also four times lower than the other two groups (NCV: 45.5%, 61/134; NV: 42.3%, 63/149, p<0.001), which was verified after adjusting age and sex (**Table 2**, **Supplementary Table 4**). The median CKMB of COVID-19 patients was 13.00 U/L (IQR: 6.38-21.25), which was similar with the NCV patients (median 11.50 U/L, IQR 1.38-25.25 U/L), but about 2 times lower than NV patients (median 25.00 U/L, IQR 17.00-37.00 U/L). Abnormal CKMB result was occurred in 17.1% (12/70) and 28.5% (53/186) of patients in COVID-19 and NCV group respectively, which had no significant difference between the two groups (p>0.05); while the proportions of the two groups were both lower than that in NV group (51.1%, 165/323, p<0.001), the multivariable logistic regressions showed consistent results (**Table 2** and **Supplementary Table 4**). In COVID-19 patients, two (9.6%), eighteen (24.3%) patients were tested with abnormal ALT and AST results respectively, and no significant difference was observed in the two tests among the three group (p>0.05) (**Table 2**). However, ALT results of COVID-19 patients (median 14.00 U/L, IQR 11.00-25.00 U/L) was lower than that in NV group (median 18.00 U/L, IQR 13.00-28.00 U/L), and the AST results of COVID-19 patients (median 31.20 U/L, IQR 21.00-40.28 U/L) was lower than that in NCV (median 36.00 U/L, IQR 30.00-46.00 U/L) and NV group (median 35.00 U/L, IQR 29.00-43.00 U/L) (**Table 2**). The comparison of COVID-19, RSV, and IFA patients further verified the results of differences and similarities between COVID-19 and NCV patients (**Supplementary Table 3**).

## Discussion

Considering the huge health risk caused by COVID-19, our assumption used to be that pediatric COVID-19 patients should present serious outcomes in comparison with other pneumonias. However, we are surprised to find out that COVID-19 pediatric pneumonia is not special and similar to other viral pneumonia. And, fortunately, majority clinical characteristics of pediatric COVID-19 patients, including clinical symptoms, signs, radiographic and laboratory tests, were milder than pneumonia caused by other pathogens. Fever and cough were the most common symptoms both in COVID-19 pneumonia and other respiratory pathogens pneumonia, while, a relatively lower proportion of COVID-19 patients presented fever, cough, sputum production, shortness of breath, abnormal pulmonary auscultation. In Li’s study, they also showed that the proportions of cough and fever symptoms in COVID-19 patients were lower than those of IFA patients, which was consist with our findings (Li et al., 2020).

The phenomenon that majority clinical characteristics among COVID-19 patients were milder might be explained by three reasons. First, it may be related to the physiological insusceptibility to SARS-CoV-2 in children. This pattern was also showed in other coronaviruses, such as SARS and MERS (Ludvigsson, 2020; Su et al., 2020). On the other hand, previous studies have also reported that angiotensin-converting enzyme 2 (ACE2) is the main biding site of SARS-CoV-2 in the lung, but the receptor is significantly less in children than adults (Zhao et al., 2020). Second, with the development of the immune system, age-dependent maturation of the immune response results in an enhanced immune function, which may protect older children from a worse disease progression caused by SARS-CoV-2 and other pathogens (Cristiani et al., 2020). In our study, patients in COVID-19 group were significantly older than patients in the other two groups. Older children’s more mature immune is a possible reason why COVID-19 patients had milder clinical characteristics. Third, pediatric COVID-19 patients were usually found through the screening for close contacts of their adult relatives with suspected SARS-CoV-2 infection. Early detection of the pediatric COVID-19 patients, as well as providing appropriate treatments (i.e. anti-viral treatment, etc.) as early as possible according to patients’ clinical conditions might avoid the disease getting worse.

In terms of CT imaging, nearly half of the pediatric COVID-19 patients presented GGO (42.5%), which was similar with the proportion of GGO (42.1%) reported among Wuhan patients (Li et al., 2020). Current study showed that the GGO was more common in COVID-19 children and consolidation was more common in IFA children, however, we did not find such differences in this study (Li et al., 2020). Another finding was that the COVID-19 patients were less likely to affect both lungs comparing with other respiratory pathogens pneumonia patients. Unilateral lung pneumonia’s relatively milder clinical characteristics proved in this study could also be a potential reason for the milder impact of COVID-19 on children compare with other kinds of pneumonia. However, though at the emergency period, it is understandable to perform chest CT for COVID-19 patients, radiological tests are not routine examinations for pediatric pneumonia patients with mild symptoms considering the potential harm, especially when PCR test for SARS-CoV-2 can confirmed quickly. To avoid the potential harm caused by radiological tests, lung ultrasound was used to assist in the diagnosis and assessment of pneumonia in children, which was also used in monitor COVID-19 pneumonia progression (Pereda et al., 2015; Dargent et al., 2020); however, lung ultrasound was not performed in this study, the potential role of lung ultrasound needs to be further verified.

And for laboratory test results, relatively lower CKMB, ALT, AST, and PCT levels were presented in COVID-19 patients compared with some other respiratory pathogens infected patients. Especially for PCT, the proportion of PCT abnormal patients in COVID-19 patients was more than four times lower than other kinds of pneumonia patients. Current study also reported a relative lower PCT value in COVID-19 patients compared with bacterial pneumonia patients, because PCT could be induced by bacteriotoxin but suppressed by interferon (Chen et al., 2020; Xia et al., 2020). In this study, no patients with abnormal CKMB occurred symptoms related to multisystem inflammatory syndrome in children (MIS-C) or pediatric inflammatory multisystem syndrome (PIMS), such as rash, lymphadenopathy, and shock. Previous studies have shown the racial differences in MIS-C among pediatric patients, which is less frequent in Asians (Feldstein et al., 2020; New York State Department of Health, 2020). And the diagnosis of MIS-C disease among pediatric patients with COVID-19 has not been reported in China (Lu et al., 2020). Similar with the findings of this study, other researches also showed most laboratory examination results was mostly normal or slightly increased or decreased in pediatric COVID-19 patients (She et al., 2020; ZhenDong et al., 2020).

Even though most clinical characteristics of COVID-19 patients were milder than other kinds of pneumonia, COVID-19 patients have some distinct characteristics in terms of laboratory blood cell tests. Relatively lower level of white blood cell count and lymphocyte count were observed in COVID-19 patients. In addition, compared with other pneumonia patients, the proportion of children with lymphocytopenia was also higher in COVID-19 patients. Previous study also found that white blood cell count was typically reduced with decreased lymphocyte count in COVID-19 patients (Guan et al., 2020). And Li’s study also reported COVID-19 patients presented lower level of white blood cells compared with IFA patients, which was consist with our finding (Li et al., 2020). However, the high proportion of lymphocytopenia was controversial. The possible mechanism is that SARS-CoV-2 may directly act on T lymphocytes and decrease the numbers of lymphocytes (Liu et al., 2020). And a high proportion of lymphocytopenia was also reported among adults COVID-19 patients similarly, which could support our finding (Zimmermann and Curtis, 2020). Zachariah, et al.’s study (Zachariah et al., 2020) found the similar results that lymphopenia was commonly observed at admission of pediatric COVID-19 patients from March to April, 2020 in America, which supported our findings. And recent study reported a higher lymphocyte level in COVID-19 patients compared with IFA, which was different from our finding (Li et al., 2020).Further studies were needed to verify the SARS-CoV-2’s effect on blood system and clarify possible mechanisms.

The limitation of this study is that only pediatric pneumonia patients from four provinces of China were included in this study. And the study period for only one month at the beginning of the COVID-19’s pandemic was relatively shorter and many features about COVID-19 might have not been documented. In addition, in the comparison of radiographic characteristics, a certain percentage of non-COVID-19 patients did not have chest CT scans, which brought bias to some extent. At last, lung ultrasound was not assessed in pediatric patients with pneumonia in this study. Given the important role of lung ultrasound in the diagnosis of pediatric pneumonia proved by previous studies, including COVID-19 pneumonia (Musolino et al., 2020; Buonsenso et al., 2021; Musolino et al., 2021), further studies are needed to strengthen the usage of lung ultrasound in differentiation of pediatric pneumonias. However, this is a pioneering study to explore the different aspects of pediatric COVID-19 and non-COVID-19 pneumonia in China, which could give practical evidences for the differential diagnosis of different pneumonias and the development of guidelines and policies.

In conclusion, clinical symptoms and signs COVID-19 pediatric pneumonia are relatively milder in COVID-19 pediatric pneumonia than those of other respiratory pathogens pneumonia, such as RSV and IFA. But the long-term effects of COVID-19 in children should be evaluated further.

## Data Availability Statement

The original contributions presented in the study are included in the article/**Supplementary Material**. Further inquiries can be directed to the corresponding authors.

## Ethics Statement

Ethics approval for this study was obtained from Beijing Children’s Hospital Ethics Committee (2020-K-20). Written informed consent from the participants’ legal guardian/next of kin was not required to participate in this study in accordance with the national legislation and the institutional requirements.

## Author Contributions

ZJ, TW, RMJ, AS, BX, and KS were responsible for the study design. SD, YB, YZ, YC, XW, JFL, GL, YX, JHL, NC, YS, MH, JL (17th author), HZ, CL, WQL, JL (21st author), LW, QF, JW, HS, RJ, CC, XG, MT, WL, YY, and GW-K contributed to the data collection and collation. XY, LG, LJ, XZ, and LW contributed to data cleaning. XY, LG, and XZ contributed to data analysis. ZJ, XY, LG, XZ, and BX contributed to writing the report. All authors contributed to the article and approved the submitted version.

## Funding

This study was supported by the National Natural Science Foundation of China (91546203, 91846302), CAMS Innovation Fund for Medical Sciences (CIFMS, 2019-12M-5-026; 2020-I2M-C&T-B-098). The funders had no role in study design, data collection, and analysis, decision to publish, or preparation of the manuscript.

## Conflict of Interest

The authors declare that the research was conducted in the absence of any commercial or financial relationships that could be construed as a potential conflict of interest.

## Abbreviations

COVID-19, Coronavirus Disease 2019; SARS-CoV-2, severe acute respiratory syndrome coronavirus 2; NCV, Non-COVID-19 viral; NV, Non-viral; GGO, ground-glass opacity; CT, computed tomography; WHO, World Health Organization; RT-PCR, real-time reverse-transcriptase-polymerase-chain-reaction; PA, passive agglutination; RSV, respiratory syncytial virus; HPIVs, human parainfluenza viruses; HMPV, human metapneumovirus; CoV, coronavirus; HRV, human rhinovirus; ADV, adenovirus; IFA, influenza A; IFB, influenza B; HBoV, human Bocavirus; M. pneumonia, Mycoplasma pneumonia; CRP, C-reactive protein; PCT, Procalcitonin; CKMB, Creatine kinase MB; ALT, Alanine aminotransferase; AST, Aspartate transaminase; MIS-C, multisystem inflammatory syndrome in children; PIMS, paediatric inflammatory multisystem syndrome (PIMS).
