# Supplementary material for: Comparison of Clinical Characteristics Among COVID-19 and Non-COVID-19 Pediatric Pneumonias: A Multicenter Cross-Sectional Study
Source: Front Cell Infect Microbiol. 2021 Jul 1;11:663884. doi: 10.3389/fcimb.2021.663884 (PMC8281119; doi:10.3389/fcimb.2021.663884)
Supplement: Supplementary file 1 [file DataSheet_1.docx]

**Supplementary files**


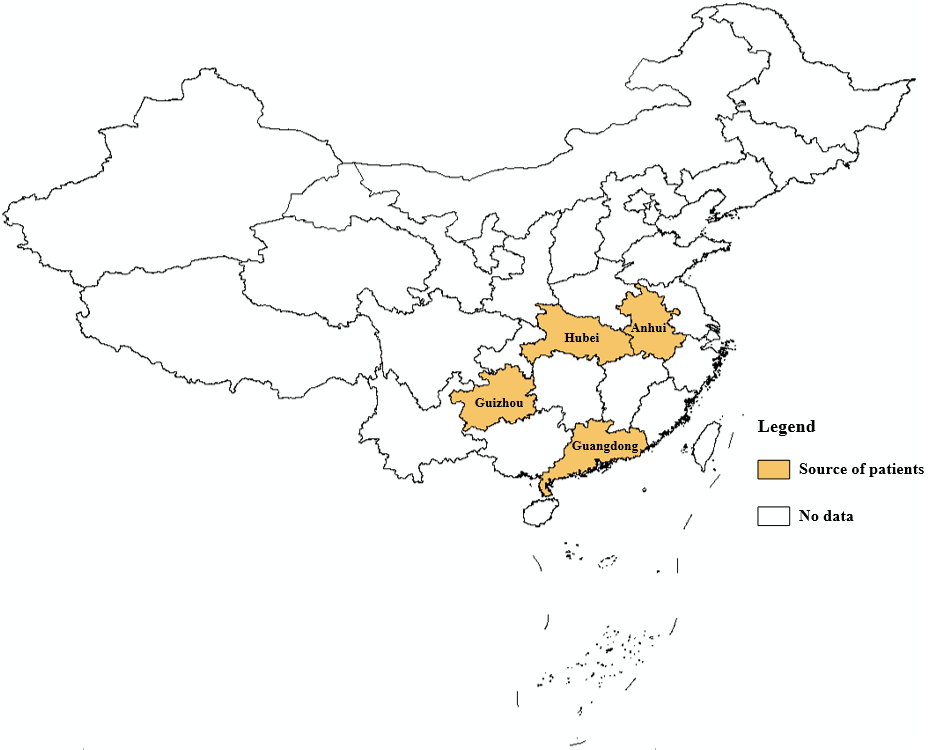


**Supplementary Figure 1. Source of pediatric pneumonia patients**

**Supplementary Table 1. Virus type of non-COVID-19 viral pneumonia patients**

| **virus type** | **number** | **percentage** |
| --- | --- | --- |
| **Single virus infection** | **164** | 84.5% |
| RSV^a^ | 59 | 30.4% |
| IFA^b^ | 31 | 16.0% |
| ADV^c^ | 20 | 10.3% |
| HRV^d^ | 17 | 8.8% |
| HPIV^e^ | 15 | 7.7% |
| HMPV^f^ | 13 | 6.7% |
| IFB^g^ | 6 | 3.1% |
| HBoV^h^ | 2 | 1.0% |
| CoV^i^ | 1 | 0.5% |
| **Mixed infection** | **30** | **15.5%** |
| RSV^a^,HBoV^h^ | 14 | 7.2% |
| HRV^d^,HPIV^e^ | 4 | 2.1% |
| HRV^d^,ADV^c^ | 2 | 1.0% |
| HBoV^h^,HMPV^f^ | 2 | 1.0% |
| HRV^d^,HMPV^f^ | 1 | 0.5% |
| IFA^b^,HMPV^f^ | 1 | 0.5% |
| HRV^d^,HBoV^h^ | 1 | 0.5% |
| CoV^i^,RSV^a^ | 1 | 0.5% |
| IFA^b^,HRV^d^ | 1 | 0.5% |
| IFA^b^,ADV^c^ | 1 | 0.5% |
| IFA^b^,ADV^c^,HRV^d^ | 1 | 0.5% |
| HRV^d^,HBoV^h^,HMPV^f^ | 1 | 0.5% |

^a^ Respiratory syncytial virus.

^b^ Influenza A virus.

^c^ Adenovirus.

^d^ Human rhinovirus.

^e^ Human parainfluenza virus.

^f^ Human metapneumovirus.

^g^ Influenza B virus.

^h^ Human Bocavirus.

^i^ Coronavirus.

**Supplementary Table 2. Demographic and clinical characteristics of patients among COVID-19 and two main types of** **non-COVID-19 viral pneumonia**

| Characteristics | | COVID-19 (N=87) | RSV^a^ (N=59) | IFA^b^ (N=31) | P value |
| --- | --- | --- | --- | --- | --- |
| **Demographics** | |  |  |  |  |
| Age (years) | |  |  |  |  |
| median(IQR) ^c,d,e^ | | 6.33(2.00-12.00) | 0.75(0.25-1.50) | 1.58(1.00-3.00) | <0.001 |
|  | <1^c,e^ | 12(13.8) | 35(59.3) | 7(22.6) | <0.001 |
|  | ≥1 to <3^d^ | 16(18.4) | 20(33.9) | 15(48.4) | 0.004 |
|  | ≥3 to <6^e^ | 13(14.9) | 3(5.1) | 7(22.6) | 0.048 |
|  | ≥6 to <12^c^ | 23(26.4) | 1(1.7) | 2(6.5) | <0.001 |
|  | ≥12^c,d^ | 23(26.4) | 0(0.0) | 0(0.0) | <0.001 |
| Female | | 49(56.3) | 22(37.3) | 12(38.7) | 0.047 |
| **Clinical symptoms and signs** | | |  |  |  |
| Fever^d^ | | 55(63.2) | 48(81.4) | 30(96.8) | <0.001 |
| Duration of Fever (days)  [median(IQR)] | | 3.00(1.00-6.00) | 5.00(2.00-7.00) | 5.00(3.00-7.00) | 0.097 |
| Cough^c,d^ | | 53(60.9) | 59(100.0) | 31(100.0) | <0.001 |
| Sputum production^c,d,e^ | | 30(34.5) | 34(57.6) | 26(83.9) | <0.001 |
| Shortness of breath^c,d^ | | 1(1.1) | 18(30.5) | 6(19.4) | <0.001 |
| Muscle ache | | 2(2.3) | 0(0.0) | 0(0.0) | 0.670 |
| Gastrointestinal symptoms | | 11/85(12.9) | 7(11.9) | 3(9.7) | 0.891 |
| Pulmonary auscultation | |  |  |  |  |
|  | Normal^c,d^ | 71(81.6) | 9(15.3) | 9(29.0) | <0.001 |
|  | Wet crackles ^c,d^ | 8(9.2) | 14(23.7) | 9(29.0) | 0.014 |
|  | Wheeze^c^ | 1(1.1) | 6(10.2) | 1(3.2) | 0.026 |
|  | Rough sound with sputum | 6(6.9) | 9(15.3) | 5(16.1) | 0.190 |
|  | Mixing abnormalities^c,d^ | 1(1.1) | 21(35.6) | 7(22.6) | <0.001 |

^a^ RSV: respiratory syncytial virus pneumonia.

^b^ IFA: influenza A virus pneumonia.

^c^ Significant difference was shown between COVID-19 group and RSV group.

^d^ Significant difference was shown between COVID-19 group and IFA group.

^e^ Significant difference was shown between RSV group and IFA group.

**Supplementary Table 3. Radiographic characteristics and laboratory findings of patients among COVID-19 and two main types of** **non-COVID-19 viral pneumonia**

| Characteristics | | COVID-19 (N=87) | RSV^a^ (N=59) | IFA^b^ (N=31) | P value |
| --- | --- | --- | --- | --- | --- |
| **Radiographic characteristics** | |  |  |  |  |
| Location | |  |  |  |  |
|  | Unilateral left lung^c^ | 18(20.7) | 2/48(4.2) | 1/23(4.3) | 0.010 |
|  | Unilateral right lung^c,e^ | 28(32.2) | 1/48(2.1) | 6/23(26.1) | <0.001 |
|  | Both lungs^c,e^ | 41(47.1) | 45/48(93.8) | 16/23(69.6) | <0.001 |
| Image changes CT scans | |  |  |  |  |
|  | GGO | 37(42.5) | 12/31(38.7) | 9/15(60.0) | 0.371 |
|  | Consolidation | 39(44.8) | 17/31(54.8) | 6/15(40.0) | 0.544 |
|  | Both changes | 11(12.6) | 2/31(6.5) | 0/15(0.0) | 0.409 |
| Pleural effusion | | 0/85(0.0) | 0/58(0.0) | 0/31(0.0) | - |
| **Laboratory test** | |  |  |  |  |
| White blood cell count | |  |  |  |  |
|  | median(IQR) [×10^9^/L]^c,d^ | 6.27(4.75-8.35) | 9.52(7.28-12.18) | 8.60(6.41-12.40) | <0.001 |
|  | Decreased | 8/85(9.4) | 2/58(3.4) | 0(0.0) | 0.118 |
|  | Increased^c,d^ | 14/85(16.5) | 26/58(44.8) | 12(38.7) | 0.001 |
| Lymphocyte count | |  |  |  |  |
|  | median(IQR) [×10^9^/L]^c,d^ | 2.48(1.74-3.96) | 4.40(2.51-6.40) | 3.96(2.31-6.54) | <0.001 |
|  | Decreased^c^ | 37/84(44.0) | 10/47(21.3) | 6/28(21.4) | 0.010 |
|  | Increased | 21/84(25.0) | 5/47(10.6) | 3/28(10.7) | 0.065 |
| CRP | |  |  |  |  |
| median(IQR) [mg/L] | | 3.37(0.81-11.48) | 4.60(0.73-15.10) | 3.40(1.13-15.18) | 0.668 |
| Abnormal | | 20/72(27.8) | 21/56(37.5) | 12/29(42.9) | 0.283 |
| PCT | |  |  |  |  |
| median(IQR) [ng/mL]^c,d^ | | 0.05(0.02-0.10) | 0.21(0.13-0.77) | 0.22(0.06-0.58) | <0.001 |
| Abnormal^c,d^ | | 6/56(10.7) | 18/37(48.6) | 12/22(54.5) | <0.001 |
| CKMB | |  |  |  |  |
| median(IQR) [U/L] | | 13.00(6.38-21.25) | 7.10(2.10-25.00) | 11.00(1.20-25.50) | 0.741 |
| Abnormal | | 12/70(17.1) | 14(23.7) | 7/29(24.1) | 0.586 |
| ALT | |  |  |  |  |
| median(IQR) [U/L]^c^ | | 14.00(11.00-25.00) | 19.00(14.00-30.75) | 17.00(11.50-33.50) | 0.016 |
| Abnormal | | 7/73(9.6) | 8/58(13.8) | 4/29(13.8) | 0.715 |
| AST | |  |  |  |  |
| median(IQR) [U/L]^c^ | | 31.20(21.00-40.28) | 37.00(28.00-48.00) | 37.00(29.00-50.00) | 0.041 |
| Abnormal | | 18/74(24.3) | 24(40.7) | 12/29(41.4) | 0.083 |

^a^ RSV: respiratory syncytial virus pneumonia.

^b^ IFA: influenza A virus pneumonia.

^c^ Significant difference was shown between COVID-19 group and RSV group.

^d^ Significant difference was shown between COVID-19 group and IFA group.

^e^ Significant difference was shown between RSV group and IFA group.

**Supplementary Table 4. Comparison of patients’ characteristics among the three pneumonia groups based on multivariable regression**

| Characteristics | | NCV ^a^ vs COVID-19 | | NV ^b^ vs COVID-19 | | NV ^b^ vs NCV ^a^ | |
| --- | --- | --- | --- | --- | --- | --- | --- |
|  |  | Adjusted OR(95%CI) ^c^ | P value | Adjusted OR(95%CI) ^c^ | P value | Adjusted OR(95%CI) ^c^ | P value |
| **Clinical symptoms and signs** **^d^** | | |  |  |  |  |  |
| Fever ^e,g^ | | 3.30(1.79-6.10) | <0.001 | 1.39(0.82-2.35) | 0.218 | 0.42(0.27-0.65) | <0.001 |
| Cough ^e,f^ | | 57.33(12.93-254.30) | <0.001 | 16.00(7.59-33.73) | <0.001 | 0.28(0.06-1.25) | 0.096 |
| Sputum production ^e,f,g^ | | 5.13(2.91-9.06) | <0.001 | 2.62(1.57-4.36) | <0.001 | 0.51(0.35-0.75) | 0.001 |
| Shortness of breath ^e,f^ | | 16.83(2.24-126.67) | 0.006 | 12.53(1.68-93.24) | 0.014 | 0.74(0.48-1.16) | 0.194 |
| Gastrointestinal symptoms | | 0.52(0.23-1.20) | 0.124 | 0.74(0.35-1.57) | 0.433 | 1.42(0.83-2.44) | 0.205 |
| Pulmonary auscultation | |  |  |  |  |  |  |
|  | Normal ^e,f,g^ | 0.09(0.05-0.18) | <0.001 | 0.05(0.03-0.10) | <0.001 | 0.54(0.34-0.86) | 0.009 |
|  | Wet crackles ^e,f^ | 6.40(2.85-14.36) | <0.001 | 9.22(4.24-20.01) | <0.001 | 1.44(0.99-2.09) | 0.054 |
|  | Wheeze | 4.27(0.53-34.63) | 0.175 | 3.89(0.50-30.33) | 0.196 | 0.91(0.41-2.00) | 0.817 |
|  | Rough sound with sputum | 1.23(0.45-3.32) | 0.689 | 1.66(0.66-4.14) | 0.280 | 1.35(0.75-2.43) | 0.313 |
|  | Mixing abnormalities ^e,f^ | 18.34(2.45-137.46) | 0.005 | 16.90(2.28-125.19) | 0.006 | 0.92(0.61-1.40) | 0.702 |
| **Radiographic characteristics** | |  |  |  |  |  |  |
| Location | |  |  |  |  |  |  |
|  | Unilateral left lung | 0.49(0.22-1.09) | 0.080 | 0.61(0.31-1.21) | 0.157 | 1.27(0.63-2.55) | 0.508 |
|  | Unilateral right lung ^e,f^ | 0.38(0.19-0.75) | 0.005 | 0.45(0.25-0.81) | 0.008 | 1.20(0.67-2.14) | 0.542 |
|  | Both lungs ^e,f^ | 3.23(1.76-5.91) | <0.001 | 2.57(1.50-4.38) | 0.001 | 0.80(0.49-1.29) | 0.351 |
| Image changes CT scans | |  |  |  |  |  |  |
|  | GGO ^g^ | 1.64(0.91-2.96) | 0.099 | 0.63(0.35-1.12) | 0.112 | 0.38(0.23-0.64) | <0.001 |
|  | Consolidation ^g^ | 0.81(0.45-1.47) | 0.493 | 1.57(0.89-2.76) | 0.119 | 1.93(1.16-3.21) | 0.012 |
|  | Both changes | 0.40(0.14-1.13) | 0.084 | 0.99(0.43-2.29) | 0.981 | 2.47(0.98-6.26) | 0.057 |
| Pleural effusion | | - | - | - | - | 0.68(0.26-1.76) | 0.426 |
| **Laboratory test** | |  |  |  |  |  |  |
| White blood cell count | |  |  |  |  |  |  |
|  | Decreased | 0.31(0.09-1.03) | 0.056 | 0.38(0.14-1.03) | 0.057 | 1.24(0.42-3.65) | 0.700 |
|  | Increased ^e,f,g^ | 3.13(1.62-6.05) | 0.001 | 5.32(2.85-9.95) | <0.001 | 1.70(1.19-2.45) | 0.004 |
| Lymphocyte count | |  |  |  |  |  |  |
|  | Decreased ^e,f^ | 0.50(0.27-0.91) | 0.023 | 0.45(0.26-0.78) | 0.004 | 0.91(0.56-1.47) | 0.692 |
|  | Increased | 1.81(0.92-3.57) | 0.087 | 1.16(0.62-2.17) | 0.635 | 0.64(0.39-1.07) | 0.089 |
| Abnormal CRP ^e,f^ | | 2.46(1.30-4.67) | 0.006 | 2.49(1.37-4.52) | 0.003 | 1.01(0.69-1.49) | 0.957 |
| Abnormal PCT ^e,f^ | | 8.15(3.15-21.11) | <0.001 | 6.83(2.68-17.41) | <0.001 | 0.84(0.52-1.36) | 0.475 |
| Abnormal CKMB ^f,g^ | | 1.12(0.53-2.34) | 0.774 | 3.38(1.69-6.75) | 0.001 | 3.03(2.01-4.57) | <0.001 |
| Abnormal ALT | | 0.42(0.15-1.18) | 0.100 | 0.47(0.18-1.23) | 0.122 | 1.11(0.57-2.18) | 0.751 |
| Abnormal AST | | 0.97(0.50-1.90) | 0.927 | 0.87(0.46-1.66) | 0.680 | 0.90(0.61-1.34) | 0.608 |

^a^ NCV: Non-COVID-19 viral pneumonia.

^b^ NV: Non-viral pneumonia.

^c^ Age and sex were adjusted in the multivariable logistic regressions. OR: odds ratio. CI: confidence interval.

^d^ In the clinical symptoms and signs, multivariable logistic regressions were not applied for muscle ache because no patients with muscle ache was reported in COVID-19 and NCV group.

^e^ Significant difference was shown between COVID-19 group and NCV group.

^f^ Significant difference was shown between COVID-19 group and NV group.

^g^ Significant difference was shown between NCV group and NV group.

**Supplementary Table 5. Impact factors of both lungs pneumonia**

| Characteristics | | Total | | COVID-19 | NCV | | NV | |
| --- | --- | --- | --- | --- | --- | --- | --- | --- |
|  |  | OR(95%CI) | AOR(95%CI) | OR(95%CI) | OR(95%CI) | AOR(95%CI) | OR(95%CI) | AOR(95%CI) |
| **Demographics** | |  |  |  |  |  |  |  |
| Age | | 0.84(0.80-0.89)^c^ | 0.92(0.85-0.99)^a^ | 0.93(0.86-1.01) | 0.85(0.69-1.03) |  | 0.81(0.74-0.90)^c^ | 0.92(0.79-1.06) |
| Sex (Female vs male) | | 0.93(0.63-1.36) |  | 0.70(0.30-1.64) | 0.82(0.38-1.78) |  | 0.85(0.48-1.49) |  |
| **Clinical symptoms and signs** | | |  |  |  |  |  |  |
| Fever | | 0.84(0.55-1.27) |  | 1.52(0.63-3.66) | 0.65(0.21-2.02) |  | 0.51(0.28-0.93)^a^ | 0.65(0.26-1.64) |
| Cough | | 2.97(1.60-5.52)^b^ | 1.28(0.52-3.19) | 1.48(0.62-3.54) | - |  | 0.71(0.15-3.43) |  |
| Sputum production | | 1.24(0.85-1.81) |  | 1.19(0.49-2.89) | 0.45(0.16-1.26) |  | 1.09(0.64-1.87) |  |
| Shortness of breath | | 3.86(2.00-7.45)^c^ | 1.83(0.78-4.34) | - | 2.66(0.87-8.07) |  | 3.10(1.34-7.19)^b^ | 1.54(0.52-4.55) |
| Muscle ache | | 0.39(0.02-6.25) |  | - | - |  | 0.34(0.02-5.50) |  |
| Gastrointestinal symptoms | | 1.18(0.67-2.09) |  | 0.98(0.28-3.50) | 2.03(0.44-9.31) |  | 1.13(0.53-2.44) |  |
| Pulmonary auscultation | |  |  |  |  |  |  |  |
|  | Wet crackles | 3.01(1.89-4.81)^c^ | 2.56(1.34-4.87)^b^ | 1.29(0.30-5.57) | 2.18(0.81-5.89) | 2.17(0.77-6.13) | 3.13(1.51-6.48)^b^ | 3.72(1.35-10.20)^a^ |
|  | Wheeze | 3.31(1.18-9.30)^a^ | 2.32(0.56-9.63) | - | 1.83(0.35-9.64) | 1.76(0.32-9.82) | 2.86(0.68-12.05) | 1.45(0.21-9.91) |
|  | Rough sound with sputum | 2.00(1.04-3.82)^a^ | 2.15(0.91-5.07) | 2.58(0.44-15.02) | 1.06(0.31-3.56) | 1.35(0.35-5.26) | 2.10(0.81-5.47) | 3.46(1.01-11.88)^a^ |
|  | Mixing abnormalities | 6.04(3.23-11.27)^a^ | 3.07(1.24-7.60)^a^ | - | 3.33(1.08-10.32)^a^ | 3.63(1.03-12.75)^a^ | 5.92(2.42-14.51)^a^ | 3.88(1.11-13.53)^a^ |
| **Radiographic characteristics** | | |  |  |  |  |  |  |
| Image changes CT scans | |  |  |  |  |  |  |  |
|  | Consolidation vs GGO | 0.76(0.47-1.22) |  | 1.73(0.69-4.31) | 0.60(0.24-1.48) |  | 0.56(0.24-1.30) |  |
|  | Both changes vs GGO | 0.91(0.41-2.02) |  | 2.88(0.71-11.62) | 0.50(0.09-2.89) |  | 0.73(0.21-2.54) |  |
| Pleural effusion | | 0.55(0.22-1.41) |  | - | 0.73(0.14-3.77) |  | 0.29(0.09-0.99)^a^ | 0.71(0.16-3.16) |
| **Laboratory test** | |  |  |  |  |  |  |  |
| White blood cell count | |  |  |  |  |  |  |  |
|  | Decreased | 0.45(0.19-1.07) | 1.30(0.36-4.69) | 0.34(0.07-1.80) | - |  | 0.51(0.14-1.85) |  |
|  | Increased | 1.59(1.08-2.34)^a^ | 1.08(0.62-1.89) | 3.42(0.98-11.94) | 0.77(0.36-1.65) |  | 1.37(0.80-2.33) |  |
| Lymphocyte count | |  |  |  |  |  |  |  |
|  | Decreased | 0.47(0.30-0.73)^b^ | 0.74(0.38-1.43) | 0.60(0.25-1.43) | 1.10(0.41-2.95) |  | 0.40(0.20-0.77)^b^ | 0.69(0.27-1.80) |
|  | Increased | 0.80(0.49-1.31) | 0.83(0.44-1.56) | 0.60(0.22-1.64) | 0.56(0.23-1.36) |  | 1.45(0.63-3.35) | 1.25(0.46-3.44) |
| Abnormal CRP | | 0.52(0.35-0.77)^b^ | 0.46(0.27-0.78)^b^ | 1.43(0.51-4.02) | 0.31(0.14-0.71)^b^ | 0.32(0.14-0.75)^b^ | 0.37(0.21-0.65)^b^ | 0.55(0.24-1.28) |
| Abnormal PCT | | 1.40(0.83-2.34) |  | 1.38(0.25-7.53) | 0.82(0.32-2.06) |  | 0.99(0.45-2.18) |  |
| Abnormal CKMB | | 1.87(1.21-2.90)^b^ | 1.09(0.60-1.97) | 1.85(0.52-6.51) | 1.35(0.51-3.57) |  | 2.01(1.13-3.56)^a^ | 0.93(0.40-2.16) |
| Abnormal ALT | | 3.01(1.16-7.82)^a^ | 5.92(1.28-27.41)^a^ | 8.67(0.99-76.14) | 0.52(0.15-1.77) |  | - |  |
| Abnormal AST | | 1.98(1.25-3.13)^b^ | 0.83(0.46-1.51) | 2.43(0.82-7.22) | 0.84(0.37-1.88) |  | 2.82(1.39-5.73)^b^ | 0.64(0.27-1.52) |

**Note:** Univariable and multivariable logistic regressions were conducted to explore the effect of clinical variables on both lungs pneumonia, which were conducted among all of these patients and in the three subgroups (COVID-19, NCV and NV), respectively. The independent variables that were significant in the univariable logistic regressions were included in the multivariable logistic regressions. Odds ratio (OR) and the 95% confidence interval was reported for the univariable logistic regression, and adjusted odds ratio (AOR) and the 95%CI was reported for the multivariable logistic regression.

In this analysis, because no significant variables in univariable logistic regressions were shown in the COVID-19 subgroup, the multivariable logistic regression was not conducted.

^a^ p<0.05

^b^ p<0.01

^c^ p<0.001

**Supplementary Table 6. Impact factors of GGO on CT scans**

| Characteristics | | Total | COVID-19 | | NCV | NV | |
| --- | --- | --- | --- | --- | --- | --- | --- |
|  |  | OR(95%CI) | OR(95%CI) | AOR(95%CI) | OR(95%CI) | OR(95%CI) | AOR(95%CI) |
| **Demographics** | |  |  |  |  |  |  |
| Age | | 0.99(0.94-1.04) | 1.12(1.03-1.22)^b^ | 1.10(1.01-1.20)^a^ | 0.97(0.80-1.17) | 0.76(0.65-0.89)^b^ | 0.77(0.61-0.98)^a^ |
| Sex (Female vs male) | | 1.48(0.97-2.27) | 1.42(0.60-3.35) |  | 2.02(0.99-4.13) | 1.20(0.58-2.50) |  |
| **Clinical symptoms and signs** | |  |  |  |  |  |  |
| Fever | | 1.29(0.81-2.05) | 0.50(0.21-1.22) |  | 1.32(0.49-3.58) | 1.60(0.76-3.35) |  |
| Cough | | 0.84(0.43-1.63) | 0.61(0.25-1.45) |  | - | 1.66(0.17-16.40) |  |
| Sputum production | | 1.16(0.75-1.80) | 0.16(0.05-0.47)^b^ | 0.18(0.06-0.55)^b^ | 6.35(2.18-18.46)^b^ | 0.98(0.47-2.07) |  |
| Shortness of breath | | 2.36(1.24-4.48)^b^ | - |  | 1.31(0.55-3.17) | 3.86(1.40-10.61)^b^ | 2.62(0.78-8.86) |
| Muscle ache | | - | - |  | - | - |  |
| Gastrointestinal symptoms | | 0.83(0.42-1.64) | 0.25(0.05-1.23) |  | 1.10(0.33-3.66) | 1.50(0.52-4.31) |  |
| Pulmonary auscultation | |  |  |  |  |  |  |
|  | Wet crackles | 0.69(0.41-1.16) | - |  | 2.03(0.80-5.14) | 0.59(0.21-1.66) |  |
|  | Wheeze | 0.28(0.08-1.03) | - |  | 0.35(0.06-1.98) | 0.33(0.03-3.31) |  |
|  | Rough sound with sputum | 0.78(0.38-1.60) | 0.21(0.02-1.85) |  | 1.06(0.31-3.61) | 1.18(0.33-4.20) |  |
|  | Mixing abnormalities | 1.70(0.90-3.18) | - |  | 1.92(0.72-5.10) | 1.90(0.59-6.11) |  |
| **Radiographic characteristics** | |  |  |  |  |  |  |
| Location | |  |  |  |  |  |  |
|  | Unilateral left vs both lungs | 0.60(0.32-1.14) | 1.93(0.63-5.95) |  | 0.34(0.10-1.21) | 0.42(0.13-1.35) |  |
|  | Unilateral right vs both lungs | 0.96(0.55-1.68) | 1.93(0.72-5.15) |  | 0.91(0.29-2.82) | 0.78(0.29-2.11) |  |
| Pleural effusion | | 0.80(0.28-2.31) | - |  | 1.04(0.22-4.85) | 0.59(0.11-3.03) |  |
| **Laboratory test** | |  |  |  |  |  |  |
| White blood cell count | |  |  |  |  |  |  |
|  | Decreased | 2.14(0.76-6.01) | 2.47(0.55-11.11) |  | - | 1.23(0.20-7.64) |  |
|  | Increased | 0.97(0.63-1.49) | 1.03(0.32-3.27) |  | 0.50(0.24-1.01) | 2.00(0.97-4.11) |  |
| Lymphocyte count | |  |  |  |  |  |  |
|  | Decreased | 0.94(0.57-1.54) | 2.28(0.94-5.52) |  | 1.06(0.44-2.56) | 0.22(0.06-0.80)^a^ | 0.39(0.09-1.76) |
|  | Increased | 0.90(0.53-1.53) | 0.59(0.21-1.65) |  | 1.53(0.64-3.68) | 0.64(0.23-1.76) | 0.76(0.25-2.38) |
| Abnormal CRP | | 1.03(0.65-1.63) | 0.98(0.34-2.82) |  | 1.05(0.49-2.22) | 1.03(0.49-2.16) |  |
| Abnormal PCT | | 1.05(0.60-1.84) | 2.55(0.43-15.20) |  | 0.83(0.35-1.97) | 0.61(0.22-1.66) |  |
| Abnormal CKMB | | 0.98(0.60-1.63) | 0.54(0.15-1.98) |  | 2.85(0.97-8.36) | 1.04(0.49-2.20) |  |
| Abnormal ALT | | 1.13(0.55-2.33) | 0.45(0.08-2.50) |  | 1.24(0.38-4.04) | 1.69(0.53-5.39) |  |
| Abnormal AST | | 0.72(0.45-1.17) | 0.39(0.12-1.22) |  | 0.83(0.40-1.72) | 0.67(0.29-1.57) |  |

**Note:** Univariable and multivariable logistic regressions were conducted to explore the effect of clinical variables on GGO, which were conducted among all of these patients and in the three subgroups (COVID-19, NCV and NV), respectively. The independent variables that were significant in the univariable logistic regressions were included in the multivariable logistic regressions. Odds ratio (OR) and the 95% confidence interval was reported for the univariable logistic regression, and adjusted odds ratio (AOR) and the 95%CI was reported for the multivariable logistic regression.

In this analysis, because only the *Shortness of breath* was significant in univariable logistic regression of the total patients, and only the *Sputum production* was significant in univariable logistic regression of the NCV subgroup, the multivariable logistic regressions were not conducted in the two groups.

^a^ p<0.05

^b^ p<0.01
